# Crystal structure of poly[*N*,*N*-diethyl-2-hy­droxy­ethan-1-aminium [μ_3_-cyanido-κ^3^ *C*:*C*:*N*-di-μ-cyanido-κ^4^ *C*:*N*-dicuprate(I)]]

**DOI:** 10.1107/S2056989016008781

**Published:** 2016-06-03

**Authors:** Peter W. R. Corfield, Emma Cleary, Joseph F. Michalski

**Affiliations:** a Department of Chemistry, Fordham University, 441 East Fordham Road, Bronx, NY 10458, USA

**Keywords:** crystal structure, copper cyanide, three-dimensional polymer

## Abstract

In the title compound, {(C_6_H_16_NO)[Cu_2_(CN)_3_]}_*n*_, the cyanide groups link the Cu^I^ atoms into an open three-dimensional anionic network, with the mol­ecular formula Cu_2_(CN)_3_^−^. One Cu^I^ atom is tetra­hedrally bound to four CN groups, and the other Cu^I^ atom is bonded to three CN groups in an approximate trigonal-planar coordination. The tetra­hedrally coordinated Cu^I^ atoms are linked into centrosymmetric dimers by the C atoms of two end-on bridging CN groups which bring the Cu^I^ atoms into close contact at 2.5171 (7) Å. Two of the cyanide groups bonded to the Cu^I^ atoms with trigonal-planar surrounding link the dimeric units into columns along the *a* axis, and the third links the columns together to form the network. The *N*,*N*-di­ethyl­ethano­lamine mol­ecules used in the synthesis have become protonated at the N atoms and are situated in cavities in the network, providing charge neutrality, with no covalent inter­actions between the cations and the anionic network.

## Abstract 2

A cyanide-bridged anionic three-dimensional network solid is described, with mol­ecular formula {Cu_2_(CN)_3_}^−^. Charge neutrality is provided by guest N-protonated *N*,*N*-di­ethyl­ethano­lamine mol­ecules.

## Chemical context   

This structure determination was undertaken as part of our ongoing study of mixed-valence copper cyanide complexes, with the goal of directed synthesis of new polymeric structures. The intention is to build amine-coordinated Cu^II^ atoms into Cu^I^ cyanide-bridged networks by having two or more CN groups coordinating to the Cu^II^ atoms as well as the amine N atoms. This has proved somewhat elusive, however. For example, in the classic mixed-valence complex Cu_3_(CN)_4_en_2_·H_2_O where en is ethyl­enedi­amine (Williams *et al.*, 1972 ▸), there is a three-dimensional Cu^I^_2_(CN)_4_^2−^ network, with coordinated Cu^II^ cations situated in cavities with no covalent links to the network. One case where a CN-linked network incorporates both Cu^I^ and Cu^II^ is that of Cu_3_(CN)_4_oen_2_, where oen is ethano­lamine (Corfield *et al.*, 1991 ▸; Jin *et al.*, 2006 ▸). Here, there are two CN groups coordinating in a *trans* configuration to Cu^II^ atoms (the resulting coordination polyhedron is distorted octa­hedral), with incorporation of Cu^II^ into the two-dimensional network. This led us to attempt a similar synthesis involving the substituted ligand dieth­yl(2-hy­droxy­eth­yl)amine, or *N*,*N*-di­ethyl­ethano­lamine, et_2_oen. Instead of the expected blue or black mixed-valence crystals, pale-yellow crystals of the title compound, (et_2_oenH)[Cu_2_(CN)_3_], were formed, in which the amine base has been protonated and does not coordinate to any Cu atom.
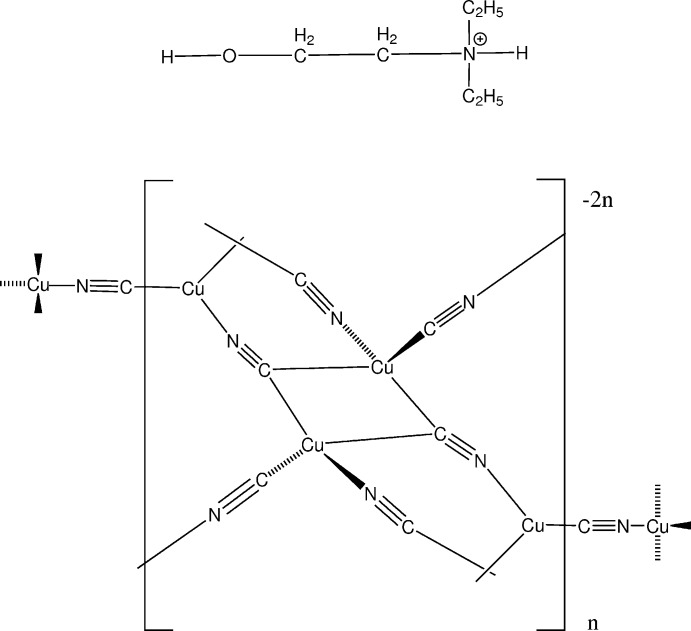


## Structural commentary   

**Figure 1:**
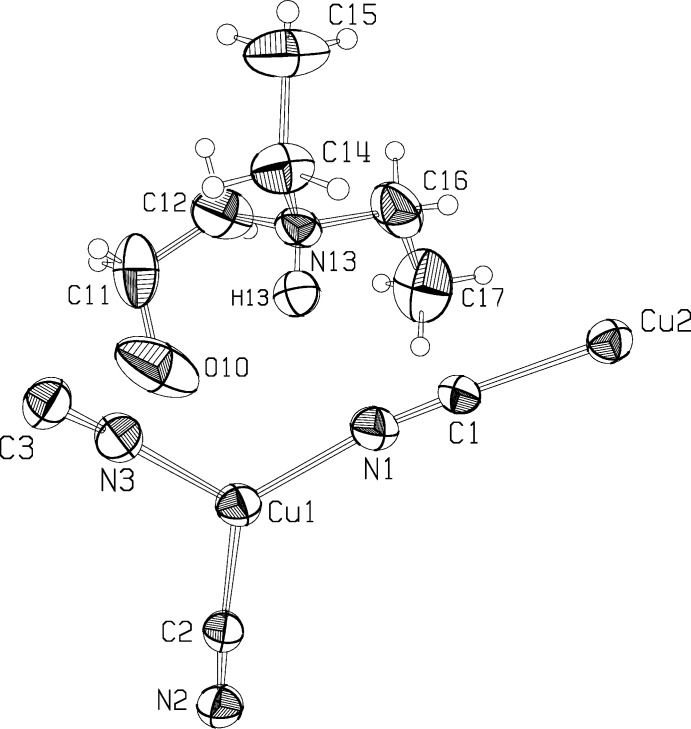
The asymmetric unit of the anionic network and of the guest cation for the title compound. Ellipsoids are drawn at the 40% probability level. Arbitrary temperature factors are used to show the H atoms, except for H13, which was refined.

**Figure 2:**
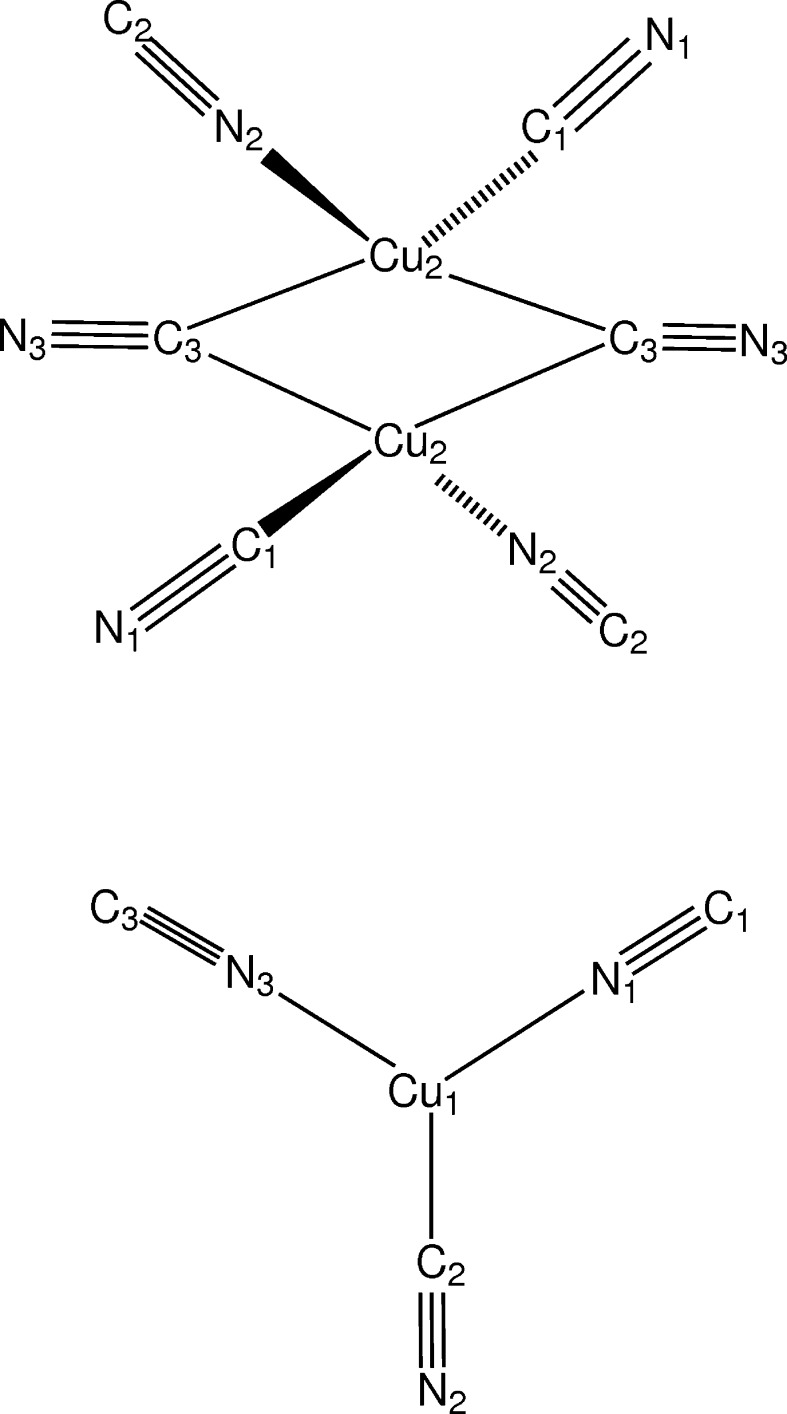
Schematic representation of the centrosymmetric Cu dimer component in the network and the trigonal Cu component.

The title compound crystallizes as a three-dimensional anionic network, [Cu_2_(CN)_3_]^−^, with the cationic protonated base occupying cavities in the network. Fig. 1 ▸ shows the structures for the asymmetric unit of the network and for the cation. The crystal structure may be considered to be built up from centrosymmetric Cu_2_(CN)_6_ dimers linked together by Cu(CN)_3_ units that are in rough trigonal–planar coordination (Fig. 2 ▸). The dimeric units are held together by two μ_3_-CN groups bonded to the dimer Cu2 atoms *via* the cyanide C atoms. There is a short Cu2⋯Cu2 distance of 2.5171 (7) Å, similar to the distance in copper metal, 2.56 Å. While there is undoubtedly some form of inter­action between the Cu2 atoms, the stereochemistry about the metal is easier to understand if the Cu⋯Cu contacts are not considered. Then the Cu^I^ atoms in the dimers are seen as bonded tetra­hedrally to four cyanide groups, two pointing away from the dimer center, and the other two bridging the two Cu^I^ atoms. Cu—C distances to the C atom of the bridging CN group are unequal, at 2.022 (3) and 2.221 (3) Å. Angles at the Cu^I^ atoms vary from 103.87 (11) to 118.03 (12) °; angles at the trigonally coordinated Cu1 atom vary from 110.73 (11) to 124.64 (11) °, and the Cu1 atom is 0.088 (2) Å from the trigonal plane through its bonded atoms, N1, C2, and N3. Selected inter­atomic distances are given in Table 1 ▸.

The cation forms a roughly spherical shape. There may be an intra­molecular hydrogen bond between the N—H bond and the hydroxyl O atom. Possible disordering in the cation is discussed below. We were not able to locate the hydroxyl H atom. The hydroxyl O atom is 2.907 (4) Å from Cu1, lying above the trigonal coordination plane in an approximately axial position. We do not consider the O atom bonded to Cu1, however.

We inter­pret the structure as a Cu^I^ complex, not the mixed-valence compound that was expected. In support of this, we cite the pale-yellow color of the compound, and also the silence in the electron spin resonance (esr) measurement (Bender, 2015 ▸). This inter­pretation requires the amine base to be protonated, for charge balance. There is indeed very clear evidence for protonation of the base N atom in the difference Fourier maps and in successful refinement of this as an unrestrained H atom. The syntheses were carried out at an initial pH of 12.4, higher than the pK_a_ of the conjugate acid of the ethano­lamine base, which we measured by titration at 9.9–10.2, depending on the ionic strength. The protonated base at this pH would be a minor component of the mixture, evidently selected by the need for charge balance as the solid polymer crystallizes.

Cu^I^ framework structures with inter­calated nitro­gen-base cations are well known [see, for example: Liu *et al.* (2005 ▸); Qin *et al.* (2011 ▸)]. Jian *et al.* (2012 ▸) describe a mixed-valence complex, {Cu^II^Cu^I^(μ-CN)_3_}_*n*_, which appears to be closely related to the present structure: it has similar unit-cell dimensions, the same space group, the same color, and the same CuCN network topology, with Cu positions close to those found here. These authors report a tri­ethyl­amine solvent mol­ecule in the network cavities. In light of the present work, we suggest that the tri­ethyl­amine mol­ecules in Jian *et al.* (2012 ▸) might be protonated. Their complex would in that case be a Cu^I^ anionic network complex similar to that reported here, rather than the mixed-valence complex they report.

## Supra­molecular features   

**Figure 3:**
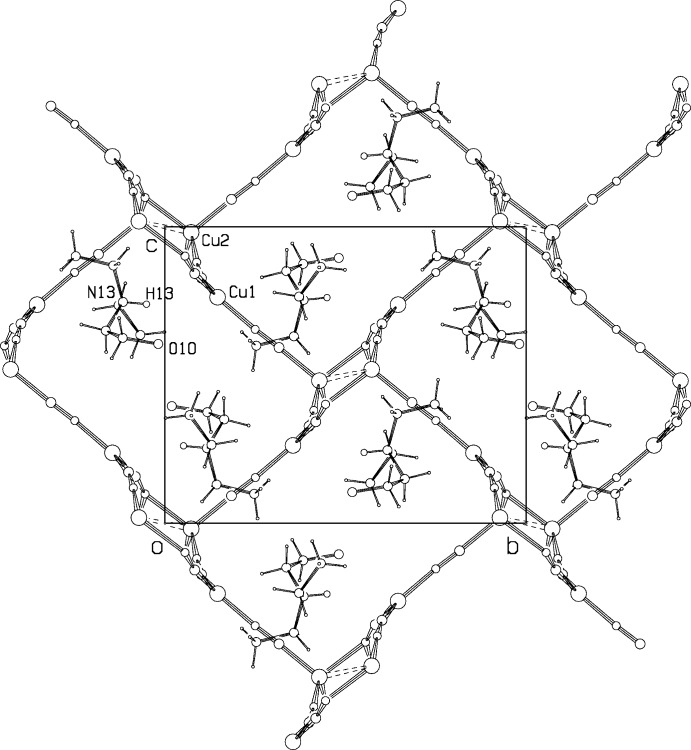
Projection of the structure down the *a* axis.

**Figure 4:**
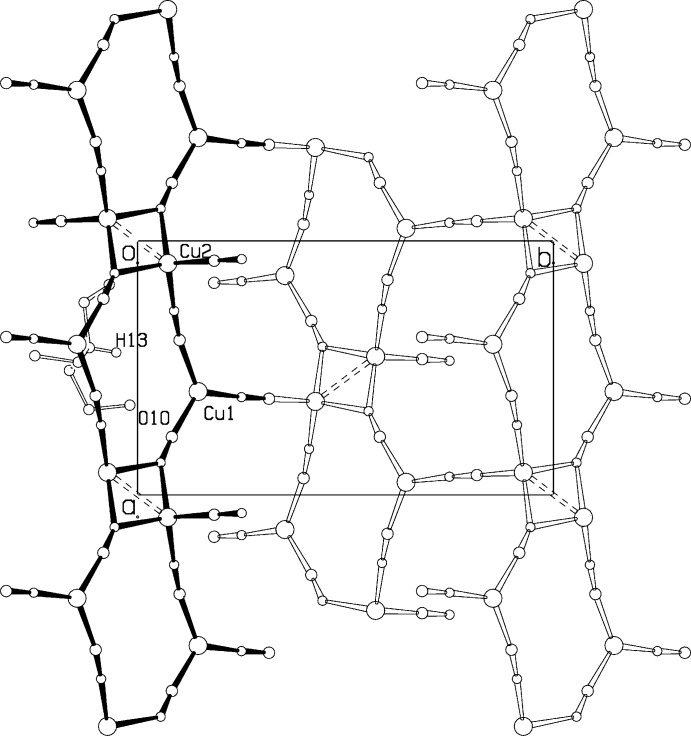
Projection of the structure down the *c* axis, showing the columns along *a*. The guest cation at (*x*,*y*,*z*) is shown, almost eclipsed by a {CuCNCu} chain.

The packing arrangement in the unit cell is shown in a projection down the *a* axis in Fig. 3 ▸, and down the *c* axis in Fig. 4 ▸. Atom Cu1 is trigonally coordinated by three CN groups, C1≡N1, C2≡N2, and C3≡N3. C1≡N1 also bonds with Cu2, one of the dimer Cu atoms, while C3≡N3 coordinates to Cu2 atoms in both a dimer at (*x*, *y*, *z*) and at (*x* + 1, *y*, *z*), thus linking the dimers into a column along the *a* axis. C2≡N2 forms a bridge to a Cu2 dimer atom related by the *n* glide plane, linking the columns into a three-dimensional network. Topology around Cu1 involves one 12-membered ring and two 18-membered rings.

There is a short contact of 3.130 (4) Å between the amine N13 and cyanide N1 atom, with H13⋯N1 = 2.35 Å and an angle N13—H13⋯N1 = 143.1°. In addition, the O10⋯N3 distance is 3.185 (5) Å. The inter­actions implied by these parameters may partially explain the overall ordering found for the CN orientations, as well as the distortions from linear geometry at N1 and N3, with Cu1—N1—C1 = 167.5 (3)° and Cu1—N3—C3 = 170.0 (3)°.

The cation hydroxide groups approach close to one another across the center of symmetry at (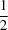, 0, 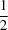), with O10⋯O10(1 − *x*, −*y*, 1 − *z*) = 2.964 (6) Å. These hydroxide groups are discussed further in the *Refinement* section.

## Database survey   

Searches of the Cambridge Structure Database (CSD, Version 5.35; Groom *et al.*, 2016 ▸) yielded 35 structures containing the Cu(CN)_2_Cu fragment with two CN groups bridging the two Cu atoms *via* the C atom. To this list we added the structures of inorganic compounds CuCN·NH_3_ (Cromer *et al.*, 1965 ▸), which contains the first example determined for this unit, and [CuCN]_3_·H_2_O (Kildea *et al.*, 1985 ▸). Cu⋯Cu distances averaged 2.53 Å, with a range of 2.31–2.69 Å. The corresponding distance in the present work is 2.5171 (7) Å, close to the observed mean. The Cu—C distances to the bridging C atom of the CN group are almost always significantly different. The shorter distance averages 2.00 Å with a limited range of 1.90–2.13 Å. The longer one ranges from 2.10 to 2.52 Å, with an average of 2.25 Å. The Cu—C distances of 2.022 (3) and 2.221 (3) Å in the present work again fall very close to these averages. There is a rough correlation between the Cu⋯Cu distance and the longer Cu—C distance, as noted by Stocker *et al.* (1999 ▸).

## Synthesis and crystallization   

The compound studied was synthesized as follows: CuCN (23 mmol) and NaCN (39 mmol) were stirred in 8 ml of water until all solids dissolved. 40 mmol of *N*,*N*-(di­ethyl­amino)­ethanol in 6 ml of water were added. The solution turned orange and slow evaporation yielded yellow crystals after several days (a green powder was also obtained in some preparations). We also prepared the compound by reduction of Cu^II^: 2 mmol CuSO_4_·5H_2_O and 40 mmol *N*,*N*-(di­ethyl­amino)­ethanol were dissolved in 15 ml of water, and 5 mmol of NaCN in 10 ml water were added. Needle-like crystals up to 2 mm long were yielded through slow evaporation.

Infra-red spectra obtained with both a Nicolet iS50 FT–IR and a Buck 550 machine showed three bands in the CN stretching region, with bands at 2072, 2099, and 2122 cm^−1^. In addition, there is a strong, broad band at 3430 cm^−1^, reflecting the presence of the OH group. This band is present also in the IR spectrum of neat *N*,*N*-di­ethyl­ethano­lamine, as well as in that of the corresponding hydro­chloride salt.

A ground-up sample of the compound was shown to be esr silent (Bender, 2015 ▸), confirming the absence of Cu^II^ species in the structure.

## Refinement details   

Crystal data, data collection and structure refinement details are summarized in Table 2 ▸. Intensities of three standard reflections were measured every two h during the 114 h of data collection. A small overall decay of 2.1 (5)% in standard intensity was noted; no correction was made for this decay.

C-bound hydrogen atoms were constrained to idealized positions with C—H distances of 0.97 Å for CH_2_ groups and 0.96 Å for CH_3_ groups, and *U*_eq_ values fixed at 1.2 times the *U*_iso_ of their bonded C atoms. The methyl torsional angles were refined. The N-bound hydrogen atom was independently refined.

After convergence in initial refinements, we observed considerable anisotropy in the displacement ellipsoid for O10, in the substituted ethano­lamine cation, indicating a possible disorder. This disorder hindered unambiguous detection of the hydroxyl H atom in difference Fourier maps. We have made extensive attempts to model the disorder without success. The models invariably led to poor geometry without improving the agreement between calculated and observed structure factors. If the geometry was restrained to reasonable values, the agreement became even poorer. Refinements of non-centric models were also carried out in light of the close approach between hydroxyl groups related by the center of symmetry at (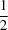, 0, 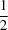). These were also unsuccessful. In an attempt to improve the electron density around the hydroxyl group, the intensity data were smoothed by a 12 parameter model with *XABS2* (Parkin *et al.*, 1995 ▸). The smoothing did improve the electron density and lowered the *R*-factor slightly, but did not improve refinements of the disordered models. The final model does not include any disorder in the cation.

The cyanide groups are mainly ordered, as indicated by refinement of C and N occupancy factors. Results clearly indicated that C3 bridges the two Cu2 atoms, not N3, and C3≡N3 was refined as ordered. Refined occupancies for the other cyanide groups were 77.8(1.4)% for C1≡N1 and 89.7(1.4)% for C2≡N2, indicating a favored orientation. Although these occupancies were significantly different from 100%, we chose to use ordered cyanide groups in our final model.

## Supplementary Material

Crystal structure: contains datablock(s) I. DOI: 10.1107/S2056989016008781/wm5295sup1.cif

Structure factors: contains datablock(s) I. DOI: 10.1107/S2056989016008781/wm5295Isup2.hkl

CCDC reference: 1482622

Additional supporting information: crystallographic information; 3D view; checkCIF report

## Figures and Tables

**Table 1:** Selected bond lengths (Å)

Cu1—C2	1.892 (3)	Cu2—C3^ii^	2.022 (3)
Cu1—N1	1.946 (3)	Cu2—C3^iii^	2.221 (3)
Cu1—N3	1.945 (2)	N1—C1	1.151 (4)
Cu2—C1	1.944 (3)	N2—C2	1.141 (4)
Cu2—N2^i^	1.986 (2)	N3—C3	1.135 (4)

Symmetry codes: (i) 
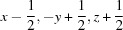
; (ii) 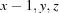; (iii) 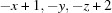.

**Table 2:** Experimental details

Crystal data	
Chemical formula	(C_6_H_16_NO)[Cu_2_(CN)_3_]
*M*_r_	323.34
Crystal system, space group	Monoclinic, *P*2_1_/*n*
Temperature (K)	298
*a*, *b*, *c* (Å)	8.3560 (11), 13.7347 (13), 11.2928 (12)
β (°)	93.991 (9)
*V* (Å^3^)	1292.9 (3)
*Z*	4
Radiation type	Mo *K*α
μ (mm^−1^)	3.27
Crystal size (mm)	0.5 × 0.3 × 0.3

Data collection	
Diffractometer	Enraf–Nonius CAD-4
Absorption correction	Gaussian (Busing & Levy, 1957 ▸)
*T*_min_, *T*_max_	0.404, 0.548
No. of measured, independent and observed [*I* > 2σ(*I*)] reflections	7241, 2534, 2160
*R*_int_	0.029
(sin θ/λ)_max_ (Å^−1^)	0.616

Refinement	
*R*[*F* ^2^ > 2σ(*F* ^2^)], *wR*(*F* ^2^), *S*	0.027, 0.082, 1.05
No. of reflections	2534
No. of parameters	148
H-atom treatment	H-atom parameters constrained
Δρ_max_, Δρ_min_ (e Å^−3^)	0.41, −0.37

Computer programs: CAD-4 (Enraf–Nonius, 1994 ▸), *SHELXS97* and *SHELXL97* (Sheldrick, 2008 ▸) and *ORTEPIII* (Burnett & Johnson, 1996 ▸). Data reduction followed procedures in Corfield *et al.* (1973 ▸); data were averaged with a local version of *SORTAV* (Blessing, 1989 ▸).

## supplementary crystallographic information

### Crystal data

**Table:**

(C_6_H_16_NO)[Cu_2_(CN)_3_]	*F*(000) = 656
*M**_r_* = 323.34	*D*_x_ = 1.661 Mg m^−^^3^*D*_m_ = 1.667 (2) Mg m^−^^3^*D*_m_ measured by Flotation in 1,2-dibromopropane/1,2,3-trichloropropane mixtures. Three independent determinations were made.
Monoclinic, *P*2_1_/*n*	Mo *K*α radiation, λ = 0.71073 Å
*a* = 8.3560 (11) Å	Cell parameters from 25 reflections
*b* = 13.7347 (13) Å	θ = 7.2–21.2°
*c* = 11.2928 (12) Å	µ = 3.27 mm^−^^1^
β = 93.991 (9)°	*T* = 298 K
*V* = 1292.9 (3) Å^3^	Rod, pale yellow
*Z* = 4	0.5 × 0.3 × 0.3 mm

### Data collection

**Table:**

Enraf–Nonius CAD-4 diffractometer	2160 reflections with *I* > 2σ(*I*)
Radiation source: fine-focus sealed tube	*R*_int_ = 0.029
Oriented graphite 200 reflection monochromator	θ_max_ = 26.0°, θ_min_ = 2.3°
θ/2θ scans	*h* = 0→10
Absorption correction: gaussian (Busing & Levy, 1957)	*k* = 0→16
*T*_min_ = 0.404, *T*_max_ = 0.548	*l* = −13→13
7241 measured reflections	3 standard reflections every 120 min
2534 independent reflections	intensity decay: −2.1(5)

### Refinement

**Table:**

Refinement on *F*^2^	Primary atom site location: heavy-atom method
Least-squares matrix: full	Secondary atom site location: difference Fourier map
*R*[*F*^2^ > 2σ(*F*^2^)] = 0.027	Hydrogen site location: inferred from neighbouring sites
*wR*(*F*^2^) = 0.082	H-atom parameters constrained
*S* = 1.05	*w* = 1/[σ^2^(*F*_o_^2^) + 0.370*P*] where *P* = (*F*_o_^2^ + 2*F*_c_^2^)/3
2534 reflections	(Δ/σ)_max_ = 0.001
148 parameters	Δρ_max_ = 0.41 e Å^−^^3^
0 restraints	Δρ_min_ = −0.37 e Å^−^^3^

### Special details

**Table:**

Geometry. All esds (except the esd in the dihedral angle between two l.s. planes) are estimated using the full covariance matrix. The cell esds are taken into account individually in the estimation of esds in distances, angles and torsion angles; correlations between esds in cell parameters are only used when they are defined by crystal symmetry. An approximate (isotropic) treatment of cell esds is used for estimating esds involving l.s. planes.

### Fractional atomic coordinates and isotropic or equivalent isotropic displacement parameters (Å^2^)

**Table:**

	*x*	*y*	*z*	*U*_iso_*/*U*_eq_	
Cu1	0.57241 (4)	0.14558 (3)	0.76249 (3)	0.04445 (13)	
Cu2	0.08692 (4)	0.07242 (2)	0.98109 (3)	0.03959 (12)	
N1	0.3754 (3)	0.10097 (19)	0.8273 (2)	0.0507 (6)	
C1	0.2650 (3)	0.08775 (19)	0.8807 (2)	0.0375 (6)	
N2	0.5860 (3)	0.31586 (18)	0.5927 (2)	0.0477 (6)	
C2	0.5850 (3)	0.2498 (2)	0.6540 (2)	0.0403 (6)	
N3	0.7582 (3)	0.08232 (19)	0.8423 (2)	0.0524 (6)	
C3	0.8645 (3)	0.0553 (2)	0.9013 (3)	0.0443 (6)	
O10	0.6123 (6)	−0.0187 (3)	0.6049 (3)	0.1317 (16)	
C11	0.6313 (5)	−0.1172 (4)	0.6251 (4)	0.0935 (15)	
H11A	0.7086	−0.1272	0.6921	0.112*	
H11B	0.6733	−0.1475	0.5561	0.112*	
C12	0.4794 (7)	−0.1640 (3)	0.6495 (4)	0.0886 (15)	
H12A	0.4998	−0.2316	0.6704	0.106*	
H12B	0.4082	−0.1628	0.5779	0.106*	
N13	0.3985 (3)	−0.11628 (18)	0.7466 (2)	0.0477 (6)	
H13	0.4166	−0.0513	0.7388	0.050 (9)*	
C14	0.4660 (5)	−0.1430 (3)	0.8694 (3)	0.0690 (10)	
H14A	0.5798	−0.1284	0.8758	0.083*	
H14B	0.4154	−0.1027	0.9266	0.083*	
C15	0.4435 (8)	−0.2471 (3)	0.9010 (5)	0.1138 (19)	
H15A	0.3332	−0.2650	0.8839	0.137*	
H15B	0.4721	−0.2563	0.9840	0.137*	
H15C	0.5106	−0.2871	0.8554	0.137*	
C16	0.2193 (5)	−0.1287 (3)	0.7354 (4)	0.0782 (12)	
H16A	0.1758	−0.1098	0.8095	0.094*	
H16B	0.1940	−0.1968	0.7214	0.094*	
C17	0.1404 (6)	−0.0684 (4)	0.6354 (5)	0.1070 (18)	
H17A	0.1726	−0.0016	0.6451	0.128*	
H17B	0.0260	−0.0730	0.6368	0.128*	
H17C	0.1728	−0.0922	0.5608	0.128*	

### Atomic displacement parameters (Å^2^)

**Table:**

	*U*^11^	*U*^22^	*U*^33^	*U*^12^	*U*^13^	*U*^23^
Cu1	0.0404 (2)	0.0412 (2)	0.0514 (2)	0.00079 (14)	0.00088 (15)	0.01524 (15)
Cu2	0.0390 (2)	0.03404 (19)	0.0458 (2)	−0.00008 (13)	0.00321 (14)	−0.00375 (13)
N1	0.0452 (14)	0.0433 (13)	0.0637 (15)	−0.0007 (11)	0.0045 (12)	0.0131 (12)
C1	0.0386 (14)	0.0305 (13)	0.0442 (13)	−0.0044 (11)	0.0083 (11)	−0.0003 (10)
N2	0.0525 (14)	0.0391 (13)	0.0526 (14)	0.0017 (11)	0.0100 (11)	0.0088 (11)
C2	0.0370 (13)	0.0369 (14)	0.0476 (14)	0.0020 (11)	0.0073 (11)	0.0084 (12)
N3	0.0388 (13)	0.0529 (15)	0.0646 (16)	0.0003 (11)	−0.0026 (12)	0.0224 (13)
C3	0.0398 (15)	0.0438 (15)	0.0487 (15)	0.0063 (12)	−0.0007 (12)	−0.0005 (12)
O10	0.194 (4)	0.106 (3)	0.103 (3)	−0.061 (3)	0.068 (3)	−0.013 (2)
C11	0.063 (3)	0.139 (5)	0.082 (3)	0.010 (3)	0.025 (2)	−0.015 (3)
C12	0.130 (4)	0.058 (2)	0.084 (3)	−0.007 (2)	0.052 (3)	−0.019 (2)
N13	0.0570 (15)	0.0349 (13)	0.0524 (14)	−0.0089 (11)	0.0121 (11)	−0.0068 (10)
C14	0.091 (3)	0.054 (2)	0.062 (2)	0.0026 (19)	0.0043 (19)	0.0008 (16)
C15	0.166 (6)	0.065 (3)	0.110 (4)	0.003 (3)	0.008 (4)	0.034 (3)
C16	0.067 (2)	0.074 (3)	0.095 (3)	−0.028 (2)	0.015 (2)	−0.015 (2)
C17	0.072 (3)	0.140 (5)	0.104 (4)	−0.007 (3)	−0.028 (3)	−0.028 (3)

### Geometric parameters (Å, º)

**Table:**

Cu1—C2	1.892 (3)	C11—H11B	0.9700
Cu1—N1	1.946 (3)	C12—N13	1.480 (4)
Cu1—N3	1.945 (2)	C12—H12A	0.9700
Cu1—O10	2.907 (4)	C12—H12B	0.9700
Cu2—C1	1.944 (3)	N13—C16	1.503 (5)
Cu2—N2^i^	1.986 (2)	N13—C14	1.505 (4)
Cu2—C3^ii^	2.022 (3)	N13—H13	0.9100
Cu2—C3^iii^	2.221 (3)	C14—C15	1.488 (5)
Cu2—Cu2^iv^	2.5171 (7)	C14—H14A	0.9700
N1—C1	1.151 (4)	C14—H14B	0.9700
N2—C2	1.141 (4)	C15—H15A	0.9600
N2—Cu2^v^	1.986 (2)	C15—H15B	0.9600
N3—C3	1.135 (4)	C15—H15C	0.9600
C3—Cu2^vi^	2.022 (3)	C16—C17	1.514 (7)
C3—Cu2^iii^	2.221 (3)	C16—H16A	0.9700
O10—C11	1.380 (6)	C16—H16B	0.9700
O10—O10^vii^	2.962 (10)	C17—H17A	0.9600
C11—C12	1.465 (6)	C17—H17B	0.9600
C11—H11A	0.9700	C17—H17C	0.9600
			
C2—Cu1—N1	124.64 (11)	O10—C11—H11B	109.3
C2—Cu1—N3	124.01 (11)	C12—C11—H11B	109.3
N1—Cu1—N3	110.73 (11)	H11A—C11—H11B	107.9
C2—Cu1—O10	100.18 (11)	C11—C12—N13	113.1 (4)
N1—Cu1—O10	97.00 (11)	C11—C12—H12A	109.0
N3—Cu1—O10	79.35 (14)	N13—C12—H12A	109.0
C2—Cu1—Cu2	123.78 (8)	C11—C12—H12B	109.0
N1—Cu1—Cu2	9.50 (8)	N13—C12—H12B	109.0
N3—Cu1—Cu2	109.32 (8)	H12A—C12—H12B	107.8
O10—Cu1—Cu2	106.14 (7)	C12—N13—C16	113.0 (3)
C1—Cu2—N2^i^	108.86 (11)	C12—N13—C14	114.4 (3)
C1—Cu2—C3^ii^	118.03 (12)	C16—N13—C14	110.9 (3)
N2^i^—Cu2—C3^ii^	109.16 (11)	C12—N13—H13	105.9
C1—Cu2—C3^iii^	108.59 (11)	C16—N13—H13	105.9
N2^i^—Cu2—C3^iii^	103.87 (11)	C14—N13—H13	105.9
C3^ii^—Cu2—C3^iii^	107.39 (9)	C15—C14—N13	114.2 (4)
C1—Cu2—Cu2^iv^	131.21 (8)	C15—C14—H14A	108.7
N2^i^—Cu2—Cu2^iv^	118.37 (8)	N13—C14—H14A	108.7
C3^ii^—Cu2—Cu2^iv^	57.35 (9)	C15—C14—H14B	108.7
C3^iii^—Cu2—Cu2^iv^	50.04 (8)	N13—C14—H14B	108.7
C1—Cu2—Cu1	7.72 (8)	H14A—C14—H14B	107.6
N2^i^—Cu2—Cu1	101.38 (7)	C14—C15—H15A	109.5
C3^ii^—Cu2—Cu1	123.79 (8)	C14—C15—H15B	109.5
C3^iii^—Cu2—Cu1	109.48 (7)	H15A—C15—H15B	109.5
Cu2^iv^—Cu2—Cu1	137.808 (18)	C14—C15—H15C	109.5
C1—N1—Cu1	167.5 (3)	H15A—C15—H15C	109.5
N1—C1—Cu2	175.2 (3)	H15B—C15—H15C	109.5
C2—N2—Cu2^v^	178.0 (3)	N13—C16—C17	112.4 (3)
N2—C2—Cu1	175.6 (3)	N13—C16—H16A	109.1
C3—N3—Cu1	170.0 (3)	C17—C16—H16A	109.1
N3—C3—Cu2^vi^	151.8 (3)	N13—C16—H16B	109.1
N3—C3—Cu2^iii^	135.4 (2)	C17—C16—H16B	109.1
Cu2^vi^—C3—Cu2^iii^	72.61 (9)	H16A—C16—H16B	107.9
C11—O10—Cu1	132.6 (3)	C16—C17—H17A	109.5
C11—O10—O10^vii^	111.2 (3)	C16—C17—H17B	109.5
Cu1—O10—O10^vii^	105.2 (2)	H17A—C17—H17B	109.5
O10—C11—C12	111.7 (4)	C16—C17—H17C	109.5
O10—C11—H11A	109.3	H17A—C17—H17C	109.5
C12—C11—H11A	109.3	H17B—C17—H17C	109.5

Symmetry codes: (i) *x*−1/2, −*y*+1/2, *z*+1/2; (ii) *x*−1, *y*, *z*; (iii) −*x*+1, −*y*, −*z*+2; (iv) −*x*, −*y*, −*z*+2; (v) *x*+1/2, −*y*+1/2, *z*−1/2; (vi) *x*+1, *y*, *z*; (vii) −*x*+1, −*y*, −*z*+1.
